# Clinical significance of *CDH13* promoter methylation as a biomarker for bladder cancer: a meta-analysis

**DOI:** 10.1186/s12894-016-0171-5

**Published:** 2016-08-30

**Authors:** Feng Chen, Tao Huang, Yu Ren, Junjun Wei, Zhongguan Lou, Xue Wang, Xiaoxiao Fan, Yirun Chen, Guobin Weng, Xuping Yao

**Affiliations:** Laboratory of Kidney Carcinoma, Ningbo urology & Nephrology Hospital, Ningbo, 315040 Zhejiang China

**Keywords:** *CDH13* methylation, Bladder cancer, Screening, Biomarker

## Abstract

**Background:**

Methylation of the tumor suppressor gene H-cadherin (*CDH13*) has been reported in many cancers. However, the clinical effect of the *CDH13* methylation status of patients with bladder cancer remains to be clarified.

**Methods:**

A systematic literature search was performed to identify eligible studies in the PubMed, Embase, EBSCO, CKNI and Wanfang databases. The pooled odds ratio (OR) and the corresponding 95 % confidence interval (95 % CI) was calculated and summarized.

**Results:**

Nine eligible studies were included in the present meta-analysis consisting of a total of 1017 bladder cancer patients and 265 non-tumor controls. A significant association was found between *CDH13* methylation levels and bladder cancer (OR = 21.71, *P* < 0.001). The results of subgroup analyses based on sample type suggested that *CDH13* methylation was significantly associated with bladder cancer risk in both the tissue and the urine (OR = 53.94, *P* < 0.001; OR = 7.71, *P* < 0.001; respectively). A subgroup analysis based on ethnic population showed that the OR value of methylated *CDH13* was higher in Asians than in Caucasians (OR = 35.18, *P* < 0.001; OR = 8.86, *P* < 0.001; respectively). The relationships between *CDH13* methylation and clinicopathological features were also analyzed. A significant association was not observed between *CDH13* methylation status and gender (*P* = 0.053). Our results revealed that *CDH13* methylation was significantly associated with high-grade bladder cancer, multiple bladder cancer and muscle invasive bladder cancer (OR = 2.22, *P* < 0.001; OR = 1.45, *P* = 0.032; OR = 3.42, *P* < 0.001; respectively).

**Conclusion:**

Our study indicates that *CDH13* methylation may play an important role in the carcinogenesis, development and progression of bladder cancer. In addition, *CDH13* methylation has the potential to be a useful biomarker for bladder cancer screening in urine samples and to be a prognostic biomarker in the clinic.

**Electronic supplementary material:**

The online version of this article (doi:10.1186/s12894-016-0171-5) contains supplementary material, which is available to authorized users.

## Background

Human bladder cancer is the most common urinary system malignancy in the world. According to global cancer statistics, approximately 74,000 cases of bladder cancer will be diagnosed in the USA in 2015, leading to an estimated 16,000 deaths [1]. Bladder cancer consists of three histological and pathological types: urothelial carcinoma, squamous cell carcinoma and adenocarcinoma. Urothelial carcinoma, also known as transitional cell carcinoma (TCC), is the most common type, accounting for 90 % of all bladder cancer cases [2, 3]. Clinically, studies have shown that non-muscle invasive bladder cancer (stages Ta – T1) accounts for approximately 70–80 % of all cases, with the remainder being characterized as muscle invasive bladder cancer (stages T2–T4). Furthermore, 10–30 % of non-muscle invasive bladder cancer (NMIBC) will progress to muscle invasive bladder cancer (MIBC) [4, 5]. MIBC patients have a much worse outcome with regards to tumor recurrence and progression, with a 5-year survival rate of 25–60 % [6–8]. Thus, additional noninvasive biomarkers for the prediction and diagnosis of bladder cancer are needed in the clinic.

Epigenetic changes are early and frequent events in cancer that play an important role in carcinogenesis. DNA methylation is the most common epigenetic alteration in human cancers [9–11]. The detection of aberrantly methylated genes can be used as a diagnostic or prognostic biomarker for human cancers, especially when the aberrant methylation silences tumor suppressor genes [12–14]. The *CDH13* gene, located on 16q24, encodes a protein that belongs to the cadherin family [15]. *CDH13*, a tumor suppressor gene (TSG), is also called H-cadherin or T-cadherin and plays a pivotal role in cell–cell adhesion [16]. The expression of *CDH13* in human tumor cells can inhibit their invasive potential and markedly reduce their proliferation [17–19]. *CDH13* promoter methylation has been reported in some human cancers including bladder cancer [16].

However, the association between *CDH13* promoter methylation and bladder cancer remains to be clarified. In this study, a meta-analysis was conducted to evaluate the effect of *CDH13* methylation on the clinicopathological features of patients with bladder cancer.

## Methods

### Search strategy

A systemic literature search for studies published prior to November 16, 2015 was conducted in the PubMed, Embase, EBSCO, CKNI and Wanfang databases without any language restrictions. The following keywords and search terms were used: (CDH13 OR cadherin 13 OR H-cadherin OR T-cadherin) AND (bladder cancer OR bladder tumor OR bladder carcinoma OR bladder neoplasm) AND (methylation OR epigenetic silencing). The reference lists of the retrieved articles and reviews were then manually searched to identify potentially relevant studies.

### Inclusion criteria

The eligible studies met the following criteria: 1) the patients were diagnosed with bladder cancer based on histopathology; 2) *CDH13* methylation was evaluated in different types of samples, such as tissue, serum, plasma and urine; 3) regarding control samples by cystoscopy and histopathological confirmation, tissue samples belonged to normal tissues, while fluid samples such as serum, plasma or urine were from healthy individuals or patients with benign urological diseases; 4) the studies showed the associations between *CDH13* methylation and clinicopathological parameters, including gender (male vs female), cancer tumor/node/metastasis (TNM) stage (T2/T4 vs Ta/T1), grade (grade 3 vs grade1/2) or tumor number (multiple vs single); 5) the methylation frequency of the *CDH13* gene was sufficient for the case-control or cohort studies; 6) the studies were published in English or Chinese. The studies that were excluded did not meet our inclusion criteria. When the authors published more than one paper using the same sample data, either the most recent study or the study using the largest sample size was selected. The current meta-analysis was reported based on the Preferred Reporting Items for Systematic Reviews and Meta-Analysis (PRISMA) statement.

### Data extraction

The following pieces of information from the eligible studies were collected: first author surname, year of publication, ethnicity, histological type, types of samples, detection method, number of samples, clinicopathological parameters, gender, stage, grade, tumor number, frequency of *CDH13* methylation, etc. As a control group, our meta-analysis used non-cancerous samples including non-cancerous diseases of the bladder and normal healthy tissue, according to each individual study in the original literature. Of these studies, a tumor stage of ≤ 1 was defined as early stage, a tumor stage of ≥ 2 was defined as advanced stage, a tumor grade of ≤ 2 was defined as low-grade, and a tumor grade of 3 was defined as high-grade. The final eligible studies were independently assessed by two reviewers for the current meta-analysis.

### Data analysis

The analysis was conducted using STATA 12.0 (Stata Corporation) to evaluate the relationships between *CDH13* methylation and bladder cancer via the pooled odds ratio (OR) and the corresponding 95 % confidence interval (95 % CI). The frequency of *CDH13* methylation was analyzed according to various cancer characteristics. A statistical test for heterogeneity was performed based on the chi-square test and Q statistic [20]. If substantial heterogeneity (I^2^ ≥ 50 % or *p* < 0.1) was observed, a random-effects model was used to calculate the parameters. Otherwise, a fixed effects model assuming a lack of heterogeneity was used [21, 22]. Egger’s test was used to evaluate for possible publication bias [23]. A *p* value of less than 0.05 was considered statistically significant.

## Results

### Study characteristics

**Fig. 1:**
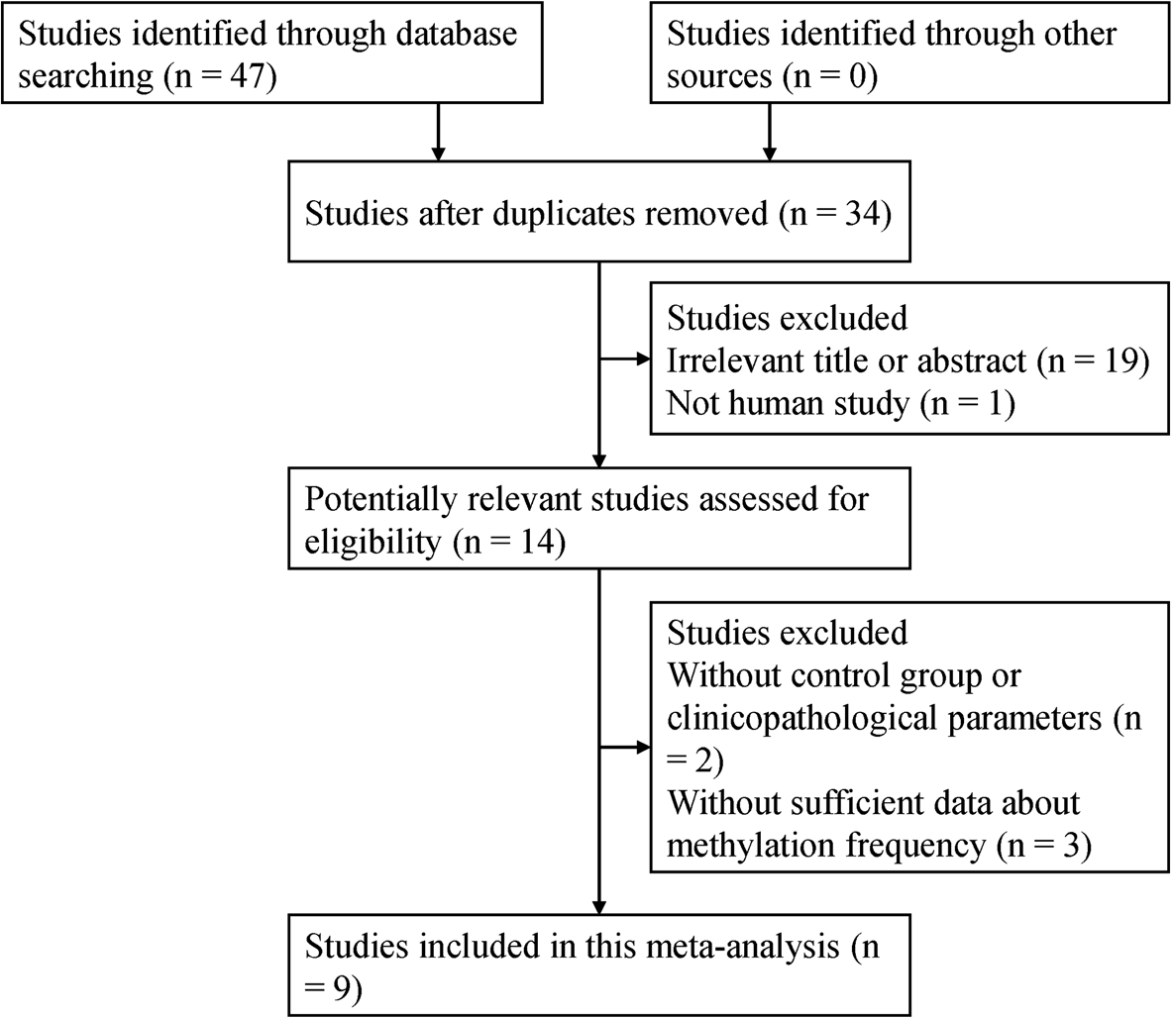
Flow diagram of the literature search strategy and the assessment of the identified studies

The search method described above obtained 49 potentially relevant articles. We carefully reviewed the titles, abstracts and full-texts of the articles. In total, 9 published studies (English, 7; Chinese, 2) met the inclusion criteria of the present meta-analysis [24–32] and included 1017 bladder cancer patients and 265 controls, as shown in Fig. 1. Of these studies, 8 studies assessed the association between *CDH13* methylation and bladder cancer risk, 5 studies evaluated the relationship between *CDH13* methylation and gender, 5 studies explored the association between *CDH13* methylation and tumor number, 4 studies reported the tumor grade (grade 3 vs grade 1-2), and 5 studies evaluated the effect of clinical stage (T2-T4 vs Ta-T1). The main characteristics of the included studies were presented in Table 1.

**Table 1:** Basic characteristics of the studies included in the current meta-analysis

					Methylation %		Number		Gender		Grade		Stage		Tumor number	
First author	Year	Ethnicity	Method	Sample	Case	Control	Cases	Controls	Male	Female	3	1-2	≥2	≤1	Multiple	Single
Maruyama et al.	2001	Caucasians	MSP	Tissue	29.9 %	-	97	-	-	-	65	32	-	-	-	-
Meng et al.	2007	Asians	MSP	Urine	15.2 %	0 %	92	30	-	-	-	-	27	65	-	-
Yu et al.	2007	Asians	MSP	Urine	16.7 %	0 %	132	30	-	-	-	-	-	-	-	-
Cabello et al.	2011	Caucasians	*	Urine	27.1 %	6 %	96	50	-	-	36	60	18	78	-	-
Agundez et al.	2011	Caucasians	*	Tissue	62.6 %	0 %	91	10	82	9	-	-	-	-	44	47
Lin et al.	2011	Asians	MSP	Serum	30.7 %	0 %	127	41	88	39	31	96	49	78	74	53
Lin et al.	2012	Asians	MSP	Tissue	35.3 %	0 %	133	43	94	39	37	96	48	85	82	51
Lin et al.	2013	Asians	MSP	Tissue	60.6 %	0 %	71	23	49	22	-	-	32	39	43	28
Lin et al.	2014	Asians	MSP	Tissue	44.9 %	0 %	178	38	124	54	-	-	-	-	76	102

*MSP* methylation-specific polymerase chain reaction, “-” indicates data not available

*indicates MS-MLPA (Methylation-Specific Multiplex Ligation-Dependent Probe Amplification)

### *CDH13* methylation and the risk of bladder caner

**Fig. 2:**
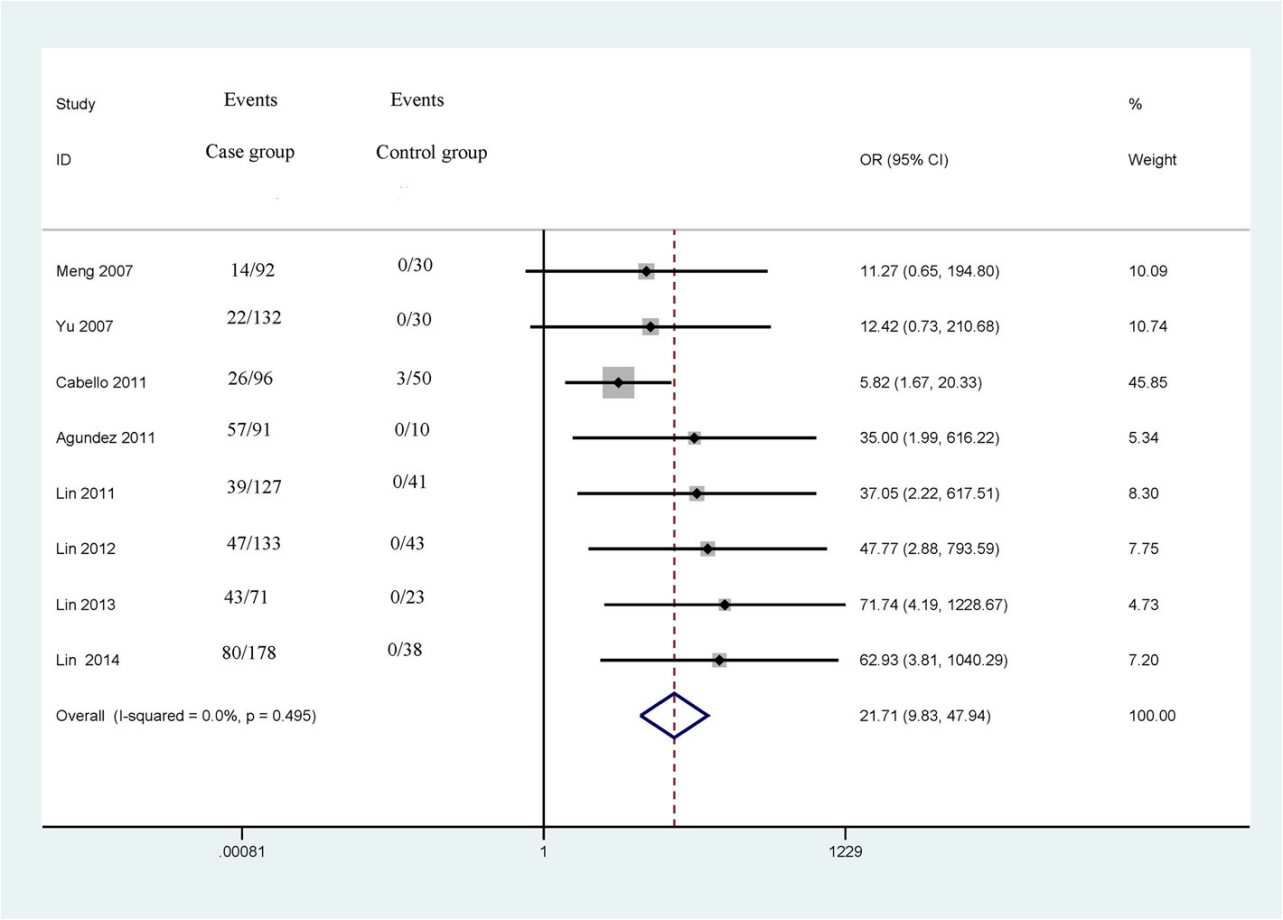
Forest plot of the association between *CDH13* methylation and bladder cancer from a fixed-effects model, including 8 studies with 920 bladder cancer patients and 265 controls, OR = 21.71, 95 % CI: 9.83–47.94, *P* < 0.001

The heterogeneity among the studies was not significant (*p* = 0.495 and I^2^ = 0.0 %), and therefore, the fixed effects model was used. The OR value of *CDH13* methylation in bladder cancer patients compared with non-tumor controls was 21.71 (95 % CI: 9.83–47.94, *P* < 0.001) (Fig. 2); this analysis included 920 bladder cancer patients and 265 controls. Subgroup analyses were performed to investigate the difference in *CDH13* methylation according to sample type (tissue and urine) and ethnicity (Caucasians and Asians) (Table 2). The results showed that the pooled OR value for the tissue group was higher than that of the urine group (OR = 53.94, 95 % CI = 12.83–226.87, *P* < 0.001; OR = 7.71, 95 % CI = 2.65–22.39, *P* < 0.001; respectively). The subgroup analysis according to ethnic populations revealed that the OR value of methylated *CDH13* in Asians was higher than in Caucasians (OR = 35.18, 95 % CI = 11.20–110.55, *P* < 0.001; OR = 8.86, 95 % CI = 2.91–27.03, *P* < 0.001; respectively).

**Table 2:** Summary of the relationship between *CDH13* methylation and bladder cancer

	Studies	Overall OR (95 % CI)	I^2^; p	*P* value	Cases	Controls	p (Egger’s test)
Total	8	21.71 (9.83–47.94)	0.0 %; 0.495	<0.001	920	265	0.008
Material:							
Urine	3	7.71 (2.65–22.39)	0.0 %; 0.831	<0.001	320	110	
Tissue	4	53.94 (12.83–226.87)	0.0 %; 0.986	<0.001	473	114	
Race:							
Caucasians	2	8.86 (2.91–27.03)	24.0 %; 0.251	<0.001	187	60	
Asians	6	35.18 (11.20–110.55)	0.0 %; 0.903	<0.001	733	205	

*CDH13* H-cadherin, *OR* odds ratio, *CI* confidence interval

### The relationships between *CDH13* methylation and clinicopathological features

**Fig. 3:**
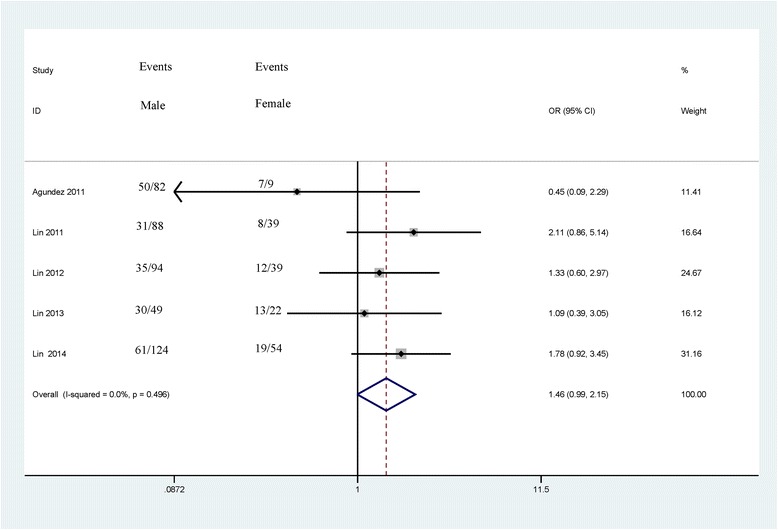
Forest plot of the association between *CDH13* methylation and gender from a fixed-effects model, including 5 studies with 437 male patients and 163 female patients, OR = 1.46, 95 % CI = 0.99–2.15, *P* = 0.053

**Fig. 4:**
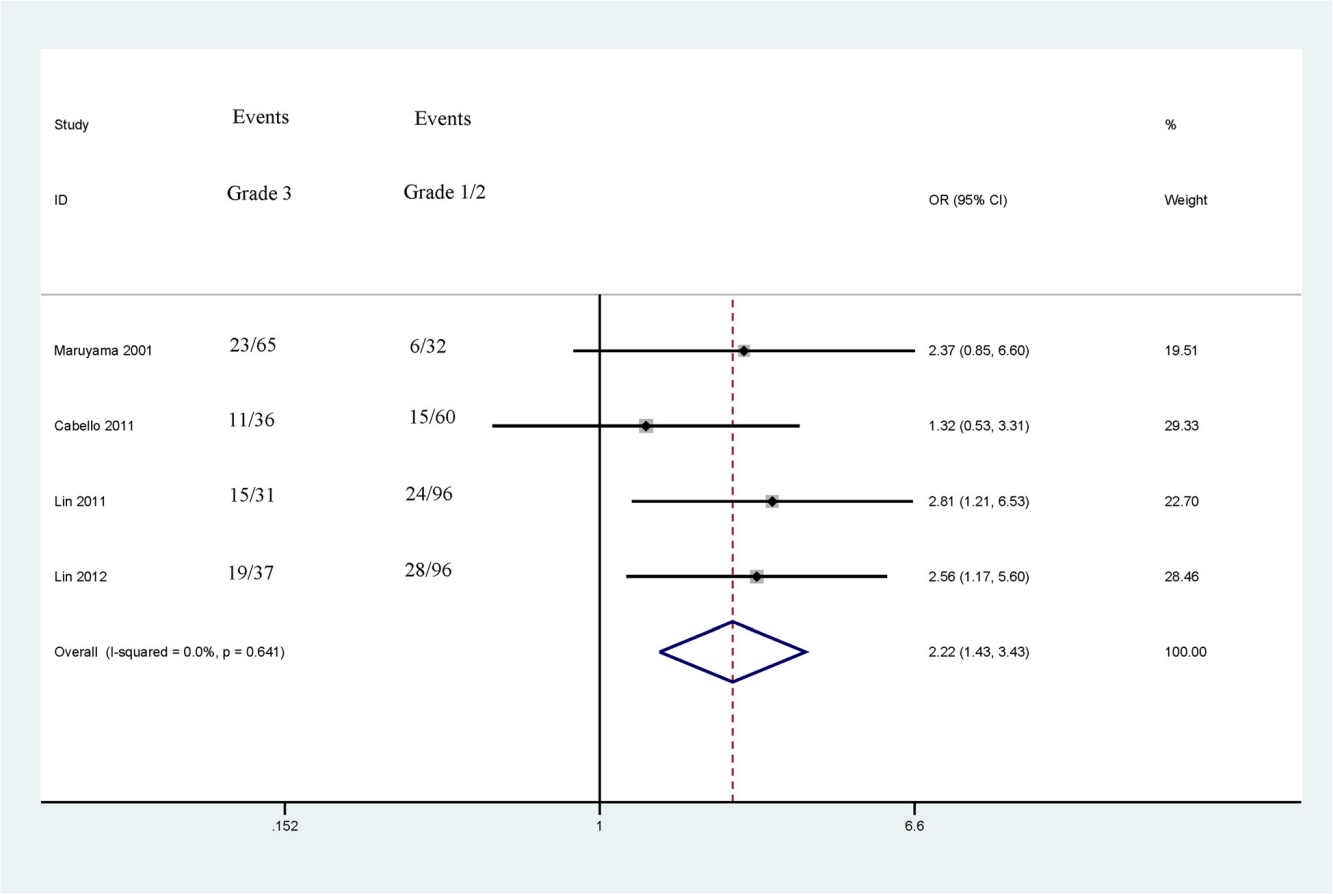
Forest plot of the association between *CDH13* methylation and tumor grade from a fixed-effects model, including 4 studies with 169 high-grade patients and 284 low-grade patients, OR = 2.22, 95 % CI = 1.72–6.80, *P* < 0.001

**Fig. 5:**
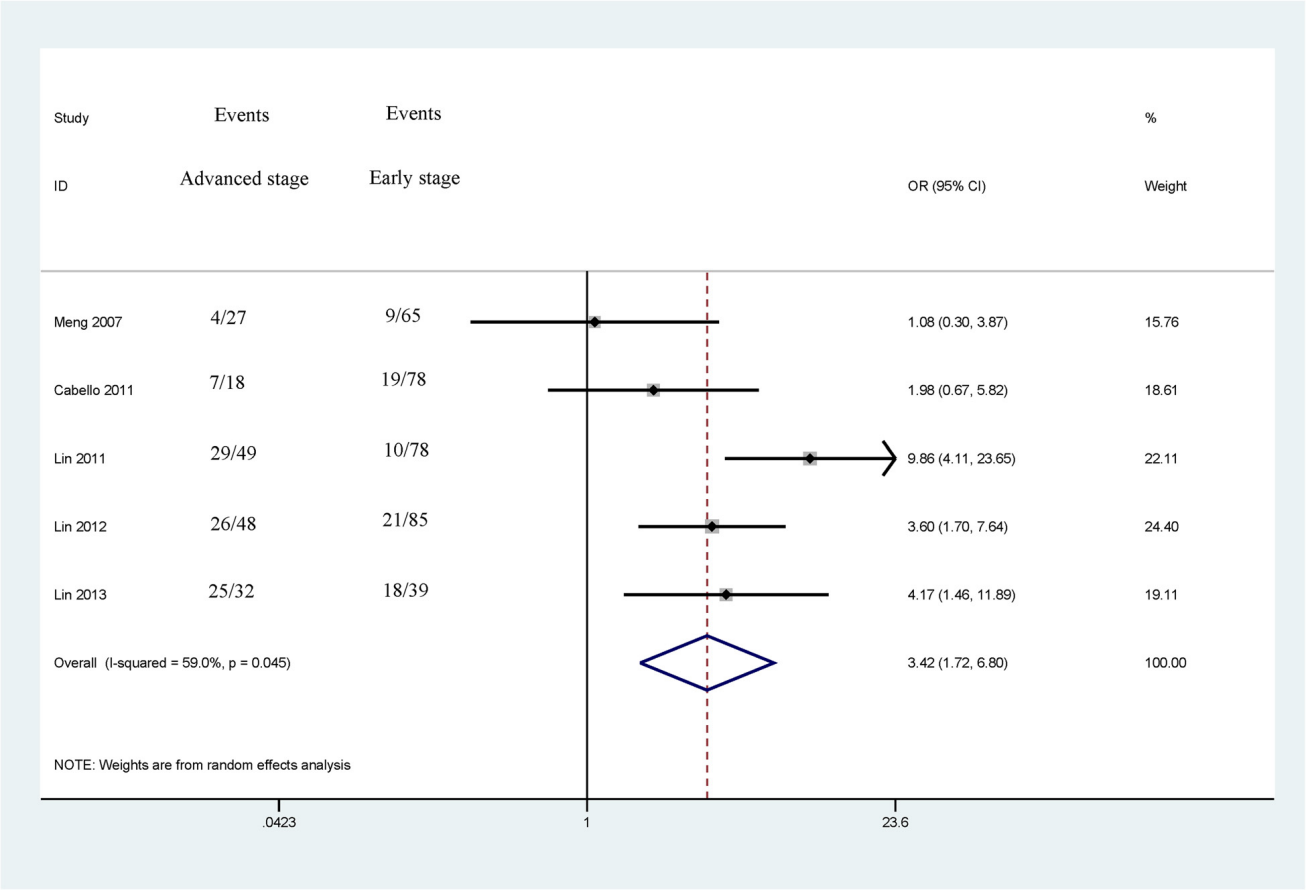
Forest plot of the correlation between *CDH13* methylation and tumor stage from a random-effects model, including 5 studies with 174 advanced bladder cancer patients and 345 early stage bladder cancer patients, OR = 3.42, 95 % CI = 1.72–6.80, *P* < 0.001

**Fig. 6:**
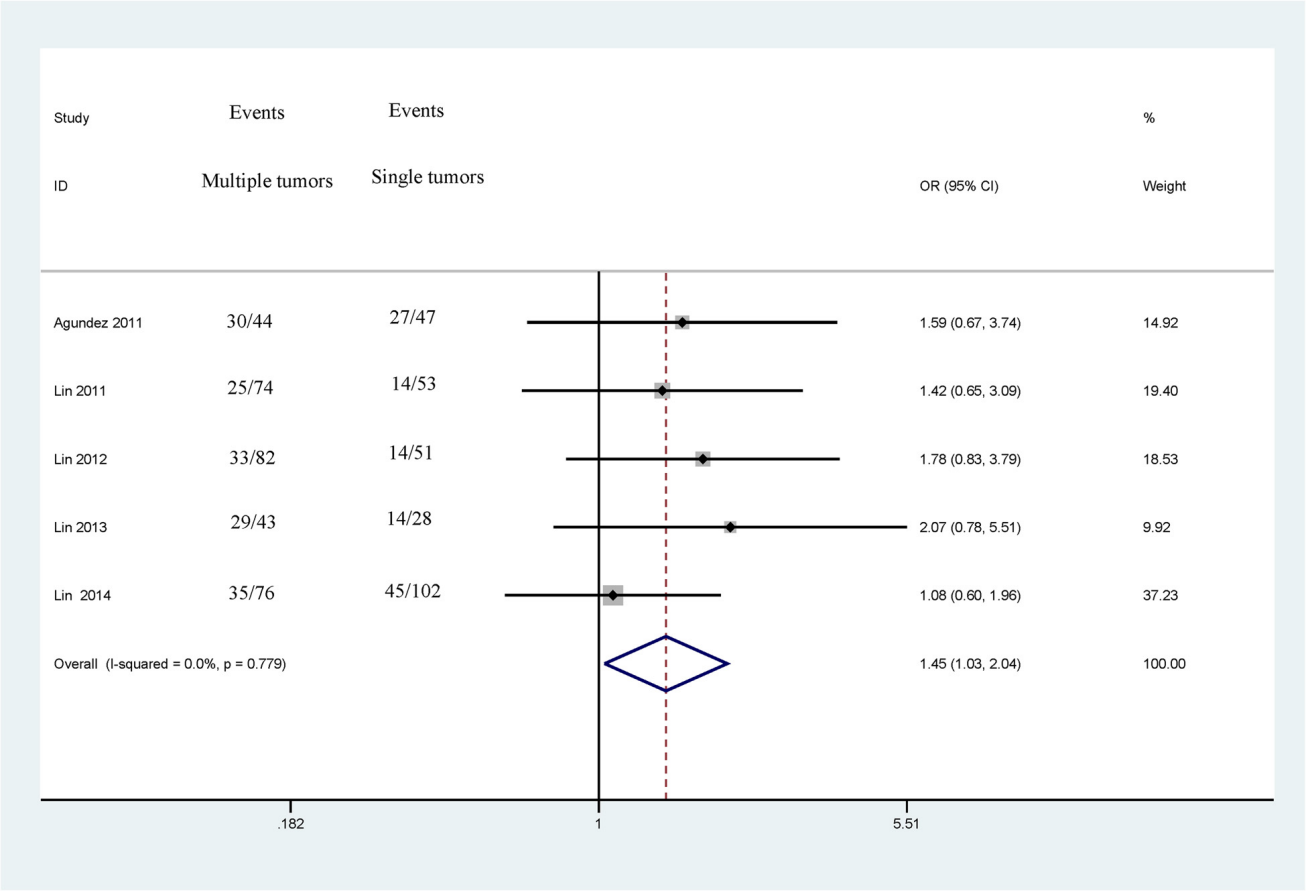
Forest plot of the correlation between *CDH13* methylation and tumor number form a fixed-effects model, including 5 studies with 319 bladder cancer patients with multiple tumors and 281 bladder cancer patients with single tumors, OR = 1.45, 95 % CI = 1.03–2.04, *P* = 0.032

The associations between *CDH13* methylation and clinicopathological features were analyzed, as shown in Table 3. The analyses of *CDH13* methylation and gender, tumor grade and tumor number used the random effects model, while a fixed effects model was used for tumor stage. A significant association was not found between *CDH13* methylation and gender in the 5 studies analyzed (OR = 1.46, 95 % CI = 0.99–2.15, *P* = 0.053), which included 437 male patients and 163 female patients (Fig. 3). The pooled OR from 5 studies including 174 advanced bladder cancer patients and 345 early stage bladder cancer patients indicated that *CDH13* methylation was significantly higher in advanced stage tumors than in early stage tumors (OR = 3.42, 95 % CI = 1.72–6.80, *P* < 0.001) (Fig. 4). Results from 4 studies comparing a total of 169 high-grade patients and 284 low-grade patients showed that *CDH13* methylation was significantly associated with high-grade bladder cancer (OR = 2.22, 95 % CI = 1.72–6.80, *P* < 0.001) (Fig. 5). Results from 5 studies analyzing a total of 319 bladder cancer patients with multiple tumors and 281 bladder cancer patients with single tumors demonstrated that methylated *CDH13* was significantly associated with patients harboring multiple tumors (OR = 1.45, 95 % CI = 1.03–2.04, *P* = 0.032) (Fig. 6).

**Table 3:** The correlations between *CDH13* methylation and clinicopathological features

	Studies	Overall OR (95 % CI)	I^2^; p	*P* value	p (Egger’s test)	Patients	
Gender						Male	Female
	5	1.46 (0.99–2.15)	0.0 %; 0.496	0.053	0.085	437	163
Grade						High-grade	Low-grade
	4	2.22 (1.43–3.43)	0.0 %; 0.641	<0.001	0.613	169	284
Stage						MIBC	NMIBC
	5	3.42 (1.72–6.80)	59.0 %; 0.045	<0.001	0.279	174	345
Number						Multiple	Single
	5	1.45 (1.03–2.04)	0.0 %; 0.779	0.032	0.038	319	281

*MIBC* muscle invasive bladder cancer (stages T2–T4), *NMIBC* non-muscle invasive bladder cancer (stages Ta – T1), *low-grade* tumor grade ≤ 2, *high-grade* tumor grade of 3

Significant heterogeneity was found in relation to tumor stage in cancer (I^2^ = 59.0 %, *p* = 0.045). Thus, a sensitivity analysis by omitting a single study was carried out to assess the stability of the pooled OR. When we removed this study by Lin 2011 et al. [25], the heterogeneity was significantly decreased, with the absence of heterogeneity (I^2^ = 14.7 %, *p* = 0.318). However, the pooled OR of *CDH13* promoter methylation was not significantly changed (OR = 2.75, 95 % = 1.71–4.44, *P* < 0.001), suggesting the stability of our analyses.

### Publication bias

Egger’s test was performed to assess for publication bias of the included studies (Tables 2 and 3). Egger’s test provided strong statistical evidence for a publication bias in the comparison between *CDH13* methylation of bladder cancer patients and non-tumor controls (*p* = 0.008). The relatively small number of control samples (265 controls versus 920 bladder cancer patients) may cause publication bias. Egger’s test indicated a lack of publication bias in the current analysis of *CDH13* methylation status and clinicopathological features (*p* > 0.05). Egger’s funnel plot of the publication bias test for *CDH13* methylation was shown in Additional file 1: Table S1.

## Discussion

DNA methylation in the blood, sputum, urine, feces, and other bodily fluids can be used as a non-invasive biomarker for the early detection of various cancers [12, 33, 34]. Aberrant methylation of the *CDH13* gene has been reported in many cancers, including non-small cell lung cancer [35], breast cancer [36], gastric cancer [37], and colorectal carcinoma [38]. However, the potential of *CDH13* gene methylation to be a biomarker for bladder cancer has not yet been evaluated. The methylation rate of *CDH13* gene was relatively lower in non-tumor control samples, with a mean methylation frequency of 1.1 % in this study. The findings of the current study showed that *CDH13* promoter methylation was significantly higher in bladder cancer patients than in non-tumor control samples (OR = 21.71, *P* < 0.001), suggesting that the methylation of *CDH13* may be involved in the development of bladder cancer. No significant heterogeneity was observed in cancer vs. controls (*p* = 0.495 and I^2^ = 0.0 %), indicating the reliability of our results. In addition, the result of a subgroup analysis based on sample type suggested that the *CDH13* methylation status was significant in both tissue and urine samples (OR = 53.94, *P* < 0.001; OR = 7.71, *P* < 0.001; respectively), indicating that the detection of *CDH13* methylation has the potential to be a non-invasive biomarker in the urine, which may aid in the early screening for and the diagnosis of bladder cancer. The OR value in Asians (OR = 35.18, *P* < 0.001) was significantly higher than in Caucasians (OR = 8.86, *P* < 0.001), which revealed that *CDH13* methylation may be a relatively more important risk factor among Asian populations.

In addition, we conducted meta-analyses to determine the correlations between *CDH13* methylation and clinicopathological characteristics. The results showed that the *CDH13* methylation status was not associated with gender (OR = 1.46, 95 % CI = 0.99–2.15, *P* = 0.053). The levels of methylated *CDH13* were significantly higher in muscle invasive bladder cancer (stages T2–T4) than in non-muscle invasive bladder cancer (stages Ta – T1) (OR = 3.42, *P* < 0.001). *CDH13* methylation status was significantly associated with high-grade (grade 3) bladder cancer (OR = 2.22, *P* < 0.001). The levels of methylated *CDH13* were significantly higher in bladder cancer consisting of multiple tumors than in bladder cancer consisting of a single tumor (OR = 1.45, *P* = 0.032). Patients with multiple tumors, high-grade bladder cancer, or muscle invasive bladder cancer are characterized by a high incidence of recurrence and progression and a poorer outcome [39, 40]. Our findings indicated that *CDH13* promoter methylation was a very useful biomarker that can predict the recurrence of bladder cancer.

Some limitations of the current meta-analysis should be considered. First, the inclusion of articles published only in English and Chinese might lead to a selection bias. Second, the primary ethnic population of the patients in the current study was Asian, while only two studies involving Caucasians were involved in this meta-analysis. Other ethnicities, such as Africans, were limited. Third, due to the limitation of eligible studies in fluid samples, we did not further evaluate the diagnostic capacity of *CDH13* promoter methylation for patients with non-muscle invasive bladder cancer. Thus, more studies based on urine and blood samples are very essential to evaluate whether *CDH13* promoter methylation can become a noninvasive biomarker for the detection and diagnosis of non-muscle invasive bladder cancer in the future. Therefore, additional studies incorporating larger sample sizes are required to confirm our results in the future.

## Conclusion

Our study indicates that *CDH13* methylation may play a key role in the initiation and progression of bladder cancer, especially among Asian populations. In addition, *CDH13* methylation has the potential to become a useful biomarker for the clinical screening of bladder cancer in the urine. *CDH13* methylation may also be a prognostic biomarker for patients with tumor progression.

## Additional file

Additional file 1: Table S1. Egger’s funnel plot of the publication bias test for *CDH13* methylation. (DOCX 21 kb)

## Abbreviations

CDH13: H-cadherin
CI: Confidence interval
MIBC: Muscle invasive bladder cancer
OR: Odds ratio
TCC: Transitional cell carcinoma
TNM: Tumor/node/metastasis.

## References

[1] Siegel RL, Miller KD, Jemal A (2015). Cancer statistics, 2015. CA Cancer J Clin.

[2] Fleshner NE, Herr HW, Stewart AK, Murphy GP, Mettlin C, Menck HR (1996). The national cancer data base report on bladder carcinoma. The american college of surgeons commission on cancer and the american cancer society. Cancer.

[3] Kantor AF, Hartge P, Hoover RN, Fraumeni JF (1988). Epidemiological characteristics of squamous cell carcinoma and adenocarcinoma of the bladder. Cancer Res.

[4] Gierth M, Burger M (2013). Bladder cancer. Progress in defining progression in nmibc. Nat Rev Urol.

[5] Jacobs BL, Lee CT, Montie JE (2010). Bladder cancer in 2010: How far have we come?. CA Cancer J Clin.

[6] Black PC, Dinney CP (2007). Bladder cancer angiogenesis and metastasis--translation from murine model to clinical trial. Cancer Metastasis Rev.

[7] Herr HW, Dotan Z, Donat SM, Bajorin DF (2007). Defining optimal therapy for muscle invasive bladder cancer. J Urol.

[8] Shariat SF, Karakiewicz PI, Palapattu GS, Lotan Y, Rogers CG, Amiel GE (2006). Outcomes of radical cystectomy for transitional cell carcinoma of the bladder: A contemporary series from the bladder cancer research consortium. J Urol.

[9] Ghavifekr Fakhr M, Farshdousti Hagh M, Shanehbandi D, Baradaran B (2013). DNA methylation pattern as important epigenetic criterion in cancer. Genet Res Int.

[10] Delpu Y, Cordelier P, Cho WC, Torrisani J (2013). DNA methylation and cancer diagnosis. Int J Mol Sci.

[11] Ma X, Wang YW, Zhang MQ, Gazdar AF (2013). DNA methylation data analysis and its application to cancer research. Epigenomics.

[12] Shivapurkar N, Gazdar AF (2010). DNA methylation based biomarkers in non-invasive cancer screening. Curr Mol Med.

[13] Kim YK, Kim WJ (2009). Epigenetic markers as promising prognosticators for bladder cancer. Int J Urol.

[14] Paluszczak J, Baer-Dubowska W (2006). Epigenetic diagnostics of cancer--the application of DNA methylation markers. J Appl Genet.

[15] Takeuchi T, Ohtsuki Y (2001). Recent progress in t-cadherin (cdh13, h-cadherin) research. Histol Histopathol.

[16] Andreeva AV, Kutuzov MA (2010). Cadherin 13 in cancer. Genes Chromosomes Cancer.

[17] Kuphal S, Martyn AC, Pedley J, Crowther LM, Bonazzi VF, Parsons PG (2009). H-cadherin expression reduces invasion of malignant melanoma. Pigment Cell Melanoma Res.

[18] Lee SW, Reimer CL, Campbell DB, Cheresh P, Duda RB, Kocher O (1998). H-cadherin expression inhibits *in vitro* invasiveness and tumor formation *in vivo*. Carcinogenesis.

[19] Lee SW (1996). H-cadherin, a novel cadherin with growth inhibitory functions and diminished expression in human breast cancer. Nat Med.

[20] Zintzaras E, Ioannidis JP (2005). Hegesma: Genome search meta-analysis and heterogeneity testing. Bioinformatics.

[21] Higgins JP, Thompson SG, Deeks JJ, Altman DG (2003). Measuring inconsistency in meta-analyses. BMJ.

[22] DerSimonian R (1996). Meta-analysis in the design and monitoring of clinical trials. Stat Med.

[23] Egger M, Davey Smith G, Schneider M, Minder C (1997). Bias in meta-analysis detected by a simple, graphical test. BMJ.

[24] Lin YL, Xie PG, Ma JG (2014). Aberrant methylation of cdh13 is a potential biomarker for predicting the recurrence and progression of non muscle invasive bladder cancer. Med Sci Monit.

[25] Lin YL, Sun G, Liu XQ, Li WP, Ma JG (2011). Clinical significance of cdh13 promoter methylation in serum samples from patients with bladder transitional cell carcinoma. J Int Med Res.

[26] Lin YL, Liu XQ, Li WP, Sun G, Zhang CT (2012). Promoter methylation of h-cadherin is a potential biomarker in patients with bladder transitional cell carcinoma. Int Urol Nephrol.

[27] Agundez M, Grau L, Palou J, Algaba F, Villavicencio H, Sanchez-Carbayo M (2011). Evaluation of the methylation status of tumour suppressor genes for predicting bacillus calmette-guerin response in patients with t1g3 high-risk bladder tumours. Eur Urol.

[28] Cabello MJ, Grau L, Franco N, Orenes E, Alvarez M, Blanca A (2011). Multiplexed methylation profiles of tumor suppressor genes in bladder cancer. J Mol Diagn.

[29] Yu J, Zhu T, Wang Z, Zhang H, Qian Z, Xu H (2007). A novel set of DNA methylation markers in urine sediments for sensitive/specific detection of bladder cancer. Clin Cancer Res.

[30] Maruyama R, Toyooka S, Toyooka KO, Harada K, Virmani AK, Zochbauer-Muller S (2001). Aberrant promoter methylation profile of bladder cancer and its relationship to clinicopathological features. Cancer Res.

[31] Meng J, Yu J, Zhu T, Zhang H, Xu H, Wang W (2007). Detection of bladder cancer by accessing DNA methylation state in urine sediments. Tumor.

[32] Lin Y, Guan T, Xiang D, Sun G, Wu G, Wang H (2013). The clinical significance of the promoter methylation of CDHl3 gene in bladder cancer. Int J Urol Nephrol.

[33] Kristiansen S, Nielsen D, Soletormos G (2014). Methylated DNA for monitoring tumor growth and regression: How do we get there?. Crit Rev Clin Lab Sci.

[34] Qureshi SA, Bashir MU, Yaqinuddin A (2010). Utility of DNA methylation markers for diagnosing cancer. Int J Surg.

[35] Drilon A, Sugita H, Sima CS, Zauderer M, Rudin CM, Kris MG (2014). A prospective study of tumor suppressor gene methylation as a prognostic biomarker in surgically resected stage i to iiia non-small-cell lung cancers. J Thorac Oncol.

[36] Moelans CB, de Groot JS, Pan X, van der Wall E, van Diest PJ (2014). Clonal intratumor heterogeneity of promoter hypermethylation in breast cancer by ms-mlpa. Mod Pathol.

[37] Tahara T, Maegawa S, Chung W, Garriga J, Jelinek J, Estecio MR (2013). Examination of whole blood DNA methylation as a potential risk marker for gastric cancer. Cancer Prev Res (Phila).

[38] Konishi K, Watanabe Y, Shen L, Guo Y, Castoro RJ, Kondo K (2011). DNA methylation profiles of primary colorectal carcinoma and matched liver metastasis. PLoS One.

[39] Lopez-Beltran A. Bladder cancer: Clinical and pathological profile. Scand J Urol Nephrol Suppl. 2008;42:95-109. 10.1080/03008880802325226 18815924

[40] Dalbagni G (2007). The management of superficial bladder cancer. Nat Clin Pract Urol.

