# Supplementary material for: Clinical significance of CDH13 promoter methylation as a biomarker for bladder cancer: a meta-analysis
Source: BMC Urol. 2016 Aug 30;16(1):52. doi: 10.1186/s12894-016-0171-5 (PMC5004266; doi:10.1186/s12894-016-0171-5)
Supplement: Additional file 1: Table S1. — Egger’s funnel plot of the publication bias test for CDH13 methylation. (DOCX 21 kb) [file 12894_2016_171_MOESM1_ESM.docx]

Egger’s funnel plot of the publication bias test for *CDH13* methylation

Egger’s funnel plot of the publication bias test for *CDH13* methylation in bladder cancer patients vs. non-tumor controls, P = 0.008.

Egger’s funnel plot of the publication bias test for *CDH13* methylation (tumor grade 3 vs. grade 1-2), P = 0.613.

Egger’s funnel plot of the publication bias test for *CDH13* methylation (stages T2-T4 vs. stages Ta-T1), P = 0.279.

Egger’s funnel plot of the publication bias test for *CDH13* methylation (male vs. female), P = 0.085.

Egger’s funnel plot of the publication bias test for *CDH13* methylation (multiple tumors vs. single tumors), P = 0.038.
